# Association of serum myokines and aerobic exercise training in patients with spinal cord injury: an observational study

**DOI:** 10.1186/s12883-016-0661-9

**Published:** 2016-08-17

**Authors:** Der-Sheng Han, Ming-Yen Hsiao, Tyng-Guey Wang, Ssu-Yuan Chen, Wei-Shiung Yang

**Affiliations:** 1 Department of Physical Medicine and Rehabilitation, National Taiwan University Hospital Beihu Branch, Taipei, Taiwan; 2 Department of Physical Medicine and Rehabilitation, National Taiwan University Hospital, Taipei, Taiwan; 3 Community and Geriatric Medicine Research Center, National Taiwan University Hospital Beihu Branch, Taipei, Taiwan; 4 Department of Internal Medicine, National Taiwan University Hospital, No. 1, Chang-Teh St, Taipei, Taiwan; 5 Research Center for Developmental Biology and Regenerative Medicine, National Taiwan University, Taipei, Taiwan

**Keywords:** Spinal cord injury, Aerobic capacity, Cardio-pulmonary exercise testing, Myostatin, Insulin-like growth factor-1

## Abstract

**Background:**

Patients with spinal cord injury (SCI) have a higher prevalence of cardiovascular diseases compared to the healthy population. Aerobic exercise training is one of the recommended treatments. However, literature regarding the effect of aerobic training on patients with SCI is scarce. This study evaluated changes in parameters of exercise physiology and serum myokines immediately after exercise and after a training program among patients with SCI.

**Methods:**

Male patients with SCI and age- and sex-matched healthy individuals were recruited. Cardio-pulmonary exercise testing (CPET) was used to determine oxygen uptake at peak exercise and anaerobic threshold in both groups. The patients with SCI attended aerobic exercise training for 36 sessions within 12–16 weeks. Basic data, hemodynamic and exercise physiology parameters, and serum myokine (myostatin, IGF-1, and follistatin) concentrations were measured pre- and post-exercise in both groups, and were repeated in patients with SCI post-training.

**Results:**

Eleven patients with SCI underwent CPET and 5 completed the training. The 11 patients and 16 healthy adults had no differences in baseline serum myokine concentrations before CPET. Immediately after the CPET, the reference group had an 18 ± 19 % increase in serum IGF-1, while the patients had no observable myokine changes. After aerobic exercise training, the 5 patients had a 48 ± 18 % increase in serum myostatin compared to the pre-training level, although the body weight and exercise physiology parameters remained unchanged.

**Conclusions:**

Acute exercise to exhaustion in CPET results in an immediate increase in serum IGF-1 in healthy individuals while aerobic exercise training results in increased serum myostatin in patients with SCI.

## Background

Over the past decades, advances in medicine and healthcare have greatly decreased risks of respiratory or urinary tract infection and renal failure in patients with spinal cord injury (SCI). However, mortality due to cardiovascular diseases continues to rise [1, 2]. There is a higher prevalence of cardiovascular diseases in patients with SCI compared to the healthy population [3, 4] partly because of decreased physical activity. Muscle weakness and atrophy, and diminished aerobic capacity are common in patients with SCI [5], while autonomic dysfunction often results in altered heart rate (HR) and blood pressure (BP) response, further heightening cardiovascular risks [6, 7].

Aerobic exercise training is the standard management for preventing and treating cardiovascular diseases [8]. It is also one of the essential components of the recommended treatments for patients with SCI [9, 10]. However, the prescription of exercise training is challenging and since most patients with SCI lack adequate balance, motor control, and muscle strength of the lower extremities, arm ergometry is a frequently used exercise modality.

Recent evidence suggests that myostatin, a strong inhibitor of muscle hypertrophy counteracting IGF-1, is also related to aerobic metabolism of muscle [11–16]. IGF-1 is a growth controlling protein that affects the development of multiple systems, with the musculo-skeletal as one of the most important [17–19]. In the hypothesis of the “accelerator-brake” model [11–14, 20], IGF-1 provides the initial muscle growth stimulus, and myostatin increases subsequently to prevent overgrowth of the muscle. Currently there are limited studies that explore the changes of serum myokines after chronic exercise training in patients with SCI. Only one small trial indicated no significant changes of serum myokines after activity-based training [21].

The present study aimed to evaluate the training effect of aerobic exercise using arm ergometry and changes in serum myokines immediately after exercise and after a training program in patients with SCI. It was hypothesized that exercise training might improve aerobic capacity and muscle strength, and result in elevated myokines.

## Methods

### Patient eligibility

Patients with SCI visiting the out-patient clinic of the Department of Physical Medicine and Rehabilitation of National Taiwan University Hospital were recruited. The inclusion criteria were age >20 years, male sex, and onset of injury more than 1 year. The diagnosis was based on the American Spinal Injury Association (ASIA) standards. Patients with known heart disease, atrial fibrillation/flutter, ventricular bigeminy, active inflammatory disease, malignancy, and cardiac pacemakers were excluded. Those who were unable to perform arm ergometry were further excluded.

For comparison of baseline serum concentration of myokines, sex- and age-matched healthy volunteers in the same facility were recruited as the reference group. We certify that all applicable institutional and governmental regulations concerning the ethical use of human volunteers were followed during the course of this research. The hospital’s Research Ethic Committee approved the study protocol and all of the participants provided written informed consent.

### Experimental protocol

Basic data, including age, height, and weight were obtained at the time of enrollment. Height was measured to the nearest 0.1 cm and weight to the nearest 0.1 kg on an electronic scale. The duration from injury to enrollment, neurologic level, and ASIA impairment scale (AIS) were recorded for the patients with SCI.

All of the participants underwent CPET. Their HR and blood BP were monitored throughout the test at rest and every 30 s. The peak responses of these parameters during CPET were also recorded. Serum myostatin, IGF-1, and follistatin concentrations were measured immediately before and after the CPET.

The patients with SCI were invited to undergo a 12- to 16-week exercise training by arm ergometry. The CPET and measurements of hemodynamic parameters and cytokines were repeated after completion of the training.

All the blood samples were taken when the participants were resting, immediately before and after the CPET and after the exercise training.

### Cardio-pulmonary exercise testing (CPET)

With the patient seated on the arm ergometer (Corival, Lode B.V., Zernikepark 16, Groningen, Netherlands) and after 2 min of baseline data collection, there was a 2 min warm-up arm cranking with no resistance before the load was increased by 10 W/min in a ramp protocol. Arm cranking was at 60–70 revolutions per min while the workload was gradually increased until volitional exhaustion or termination according to the guidelines of American College of Sports Medicine [22]. The CPET was conducted by a physiatrist and performed at least two hours after a meal.

During exercise, continuous 12-lead electrocardiogram and blood pressure (TANGO, SunTech Medical Instruments, NC, USA) were monitored. The expired air was analyzed by computerized breath-by-breath metabolic system (Quark b2 system, Cosmed s.r.l., Rome, Italy, or Cortex MetaMax 3B system, Leipzig, Germany). Exercise cardio-pulmonary parameters, including workload, minute ventilation $\left({\overset{.}{\mathrm{V}}}_{\mathrm{E}}\right)$, oxygen uptake $\left(\overset{.}{\mathrm{V}}{\mathrm{O}}_2\right)$, carbon dioxide production $\left(\overset{.}{\mathrm{V}}{\mathrm{CO}}_2\right)$, oxygen pulse, ventilator equivalent for oxygen $\left({\overset{.}{\mathrm{V}}}_{\mathrm{E}}/\overset{.}{\mathrm{V}}{\mathrm{O}}_2\right)$, and ventilatory equivalent for carbon dioxide $\left({\overset{.}{\mathrm{V}}}_{\mathrm{E}}/\overset{.}{\mathrm{V}}{\mathrm{CO}}_2\right)$, were processed.

Two independent observers with experience in CPET determined the ventilatory threshold. The anaerobic threshold (AT) was based on at least two of the following criteria: 1) ${\overset{.}{\mathrm{V}}}_{\mathrm{E}}/\overset{.}{\mathrm{V}}{\mathrm{O}}_2$ began to increase systematically without a corresponding increase in ${\overset{.}{\mathrm{V}}}_{\mathrm{E}}/\overset{.}{\mathrm{V}}{\mathrm{CO}}_2$; 2) the end tidal PO_2_ began to increase without a decrease in the end tidal PCO_2_; and 3) departure from linearity for minute ventilation. Discrepancy in the interpretation AT was solved via discussion. The peak oxygen uptake (peak $\overset{.}{\mathrm{V}}{\mathrm{O}}_2$) indicated the highest $\overset{.}{\mathrm{V}}{\mathrm{O}}_2$ value recorded using CPET and was used as the main outcome after the training period.

### Exercise training by arm ergometry

The exercise training by arm ergometry for the patients with SCI was 30 min per session, with 3 sessions per week for 12 weeks. In case of a missed session, the training duration was prolonged to a maximum of 16 weeks to complete 36 training sessions. The intensity of training was guided by CPET results, targeting an intensity of $\overset{.}{\mathrm{V}}{\mathrm{O}}_2$ at AT throughout the training program. Using a non-invasive hemodynamic monitoring system (Polar Electro OY, Finland), HR was monitored throughout the exercise and the BP every 10 min.

Interval training was adopted, with 1 min of rest between exercises. The duration of exercise progressed as follows: one min for the 1^st^ week, 2 min for the 2^nd^ week, 3 min for the 3^rd^ and 4^th^ weeks, 6 min for the 5^th^ and 6^th^ weeks, 12 min for the 7^th^ and 8^th^ weeks, 15 min for the 9^th^ and 10^th^ weeks, and 30 min for the 11^th^ and 12^th^ weeks.

### ELISA of myostatin, IGF-1, and follistatin

Serum myostatin levels were measured using competitive immunoassay kits according to the manufacturer’s protocol (Immunodiagnostik AG, Bensheim, Germany). The full-length myostatin peptide was measured with high specificity. The test sensitivity was 270 pg/ml, while the intra- and inter-assay variabilities were <10 and <15 %, respectively [23, 24].

Serum IGF-1 levels were measured using ELISA kits (Mediagnost, Reutlingen, Germany). Sensitivity was 0.09 ng/ml and the inter- and intra-assay variability were 6.8 and 6.7 %, respectively [25].

Serum follistatin was analyzed by sandwich ELISA (QuantikineH, R&D Systems, Minneapolis, MN, USA). Sensitivity was 29 pg/ml and intra- and inter-assay variability were 2.4, and 7.1 %, respectively [25].

### Statistical analysis

The optical density values versus concentration for the standard curve were fitted to a four-parameter logistic regression model. The test of differences between the SCI and reference groups was performed non-parametrically using the Mann–Whitney test, while the Wilcoxon matched pairs test was used to analyze changes in the parameters before and after exercise, or before and after training. Statistical significance was set at *p* < 0.05. Descriptive statistics (mean, standard deviation and coefficient of variation [CV]) and the statistical tests were performed using the SPSS® 11.5 (SPSS Inc. Chicago, Illinois, USA).

## Results

### Basic data

**Fig. 1:**
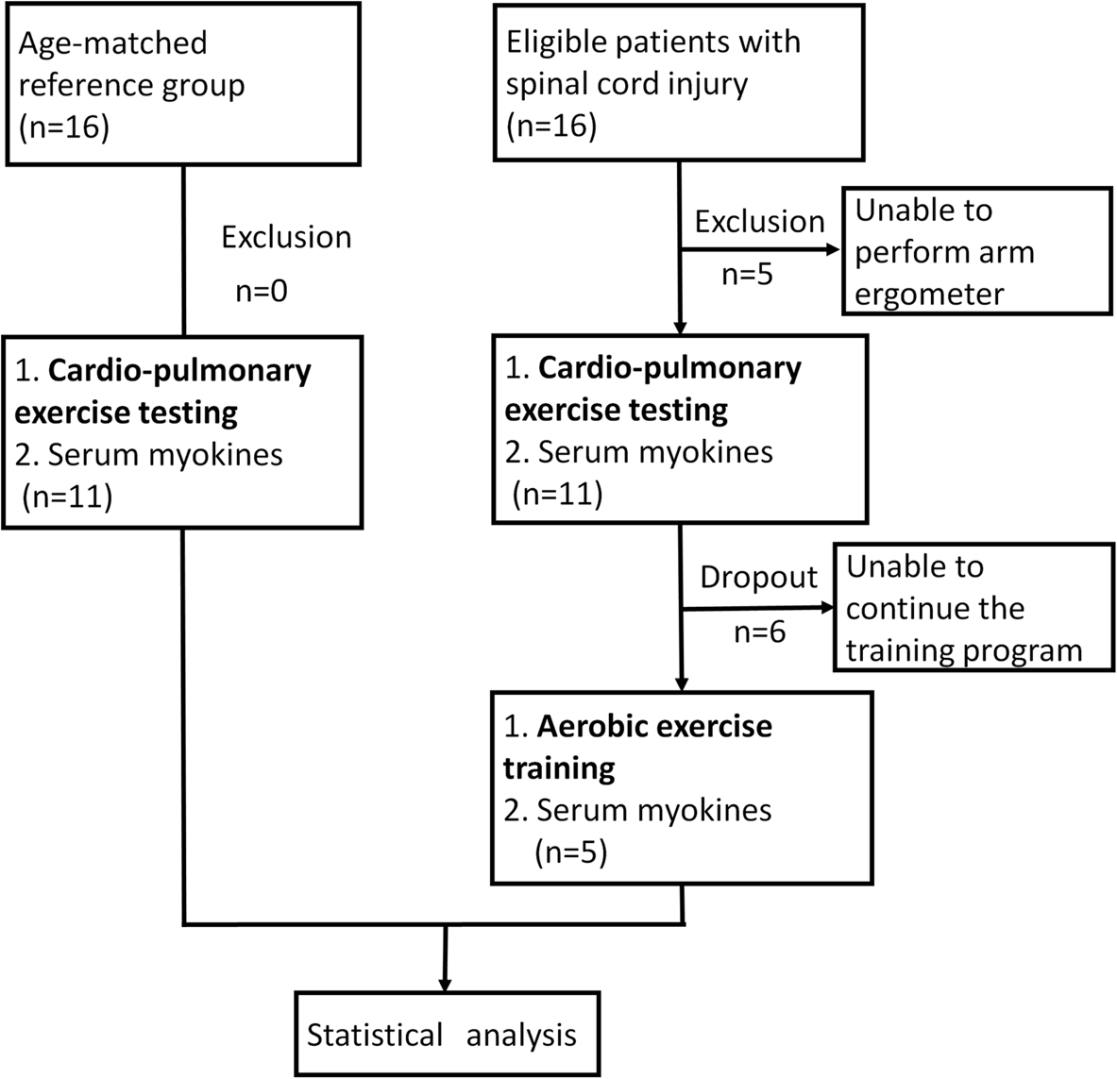
Flow diagram of experiment

There were 16 patients with SCI recruited. Five were excluded because of inability perform arm ergometry. Six patients dropped out during the exercise training program for personal reasons (Fig. 1), no adverse event was reported during the training. Table 1 lists the injury levels and the impairment scales of the patients with SCI. The mean duration from onset of injury to exercise test was 13.9 ± 5.0 years.

**Table 1:** Injury level and severity of SCI among the study patients

Patient	Bone level	Motor level	Sensory level	Neurologic level	AIS grade	Dropout
1	C3	NA	C4	C4	D	-
2	C5	C5	C5	C5	A	-
3	NA	T6	T6	T6	A	-
4	L1-2	L1	L1	L1	A	-
5	C5	C6	C5	C5	B	-
6	C4-5	C4	C4	C4	A	+
7	L4	L3	L2	L2	D	+
8	L1	L1	L1	L1	A	+
9	C4-5	C5	C4	C4	A	+
10	C5-6	C6	C6	C6	A	+
11	NA	T1	C5	C5	C	+

Abbreviations: *AIS* the American spinal injury association impairment scale; *NA* not applicable

There were 16 healthy male adults in the reference group, and no differences existed in age and body weight and height when compared with the SCI group (Table 2). There were also no differences in resting serum myokine concentrations between groups. The reference group had significantly higher heart rate, work load and oxygen uptake at peak exercise (Table 2).

**Table 2:** Basic data and change of serum myokines after exercise testing in reference and SCI (pre-training) groups

		Reference (*n* = 16)	SCI (*n* = 11)	*p* value^b^
Basic data	Age (year)	44.7 ± 6.4	39.9 ± 7.1	0.093
	Height (cm)	171.8 ± 4.6	169.7 ± 5.7	0.298
	Weight (kg)	71.9 ± 9.9	72.0 ± 11.5	0.786,
	BMI (kg/m^2^)	24.3 ± 2.4	25.0 ± 3.7	0.693
Resting	myostatin (ng/ml)	6.1 ± 1.6	6.1 ± 1.8	0.980
	IGF-1 (ng/ml)	223.4 ± 62.2	216.4 ± 49.5	0.805
	follistatin (ng/ml)	1284.4 ± 809.0	1100.9 ± 354.7	0.587
Change of myokines	Δmyostatin (ng/ml)	0.4 ± 1.6	0.3 ± 1.1	0.348
	ΔIGF-1 (ng/ml)	40.6 ± 43.0^a^	2.1 ± 48.3	0.030
	Δfollistatin (ng/ml)	59.4 ± 349.7	55.5 ± 498.4	0.657
Peak exercise	HR (beat/min)	138.8 ± 13.8	117.4 ± 23.8	0.031
	$\overset{.}{\mathrm{V}}{\mathrm{O}}_2$ (ml/min)	1454.5 ± 263.9	877.5 ± 426.2	0.012
	$\overset{.}{\mathrm{V}}{\mathrm{O}}_2$ (ml/min/kg)	20.5 ± 4.0	12.3 ± 5.5	0.026
	${\overset{.}{\mathrm{V}}}_{\mathrm{E}}$ (ml/min)	63912.5 ± 15181.1	32845.5 ± 15628.5	0.032
	Work load (watt)	38.0 ± 31.0	80.0 ± 19.0	0.009

Abbreviations: *SCI* spinal cord injury, *BMI* body mass index, IGF-1 insulin-like growth factor-1, HR heart rate, *VO*_*2*_ oxygen uptake, *V*_*E*_ minute ventilation

^a^ *p* < 0.05 by comparison between resting and peak exercise status by Wilcoxon matched pairs test

^b^Comparison between reference and SCI groups by Mann–Whitney test

### Cardio-pulmonary exercise testing (CPET)

Table 2 lists the baseline values and changes of concentrations of myokines after CPET. The reference group had significantly increased IGF-1 after CPET (18 ± 19 % above baseline, *p* = 0.001), while myostatin and follistatin remained unchanged (*p* = 0.179 and 0.918, respectively). The serum myokine concentrations in the SCI group after CPET showed no differences when compared with the values before CPET (*p* = 0.594, 0.772, 0.504, for myostatin, IGF-1, and follistatin, respectively)..

### Exercise training by arm ergometry

Five patients with SCI completed the 12–16 weeks of exercise training. After training, serum myostatin concentration increased by 48 ± 18 %, which was significantly higher compared to the pre-training status (Table 3). IGF-1 and follistatin remained unchanged. The increase in peak $\overset{.}{\mathrm{V}}{\mathrm{O}}_2$ and peak ${\overset{.}{\mathrm{V}}}_{\mathrm{E}}$ were not significant. The body weight and resting and peak hemodynamic parameters also remained the same. Two of the five patients did not reach AT $\overset{.}{\mathrm{V}}{\mathrm{O}}_2$ during CPET due to fatigue.

**Table 3:** The comparison in the SCI groups before and after 12–16 weeks of training

		SCI (*n* = 5)		*P* value^a^
		Pre-training	Post-training	
Basic data	Age (year)	39.6 ± 7.4	-	-
	Height (cm)	167.6 ± 6.0	167.6 ± 6.0	-
	Weight (kg)	65.6 ± 6.6	67.9 ± 7.3	0.180
	BMI (kg/m^2^)	23.5 ± 3.4	24.3 ± 3.8	0.180
Resting	HR (beat/min)	72.5 ± 13.8	71.8 ± 13.8	0.992
	SBP (mmHg)	109.5 ± 24.8	110.6 ± 32.1	0.128
	DBP (mmHg)	65.9 ± 11.6	67.4 ± 15.0	0.799
Peak exercise	HR (beat/min)	117.4 ± 23.8	120.4 ± 19.7	0.866
	SBP (mmHg)	136.8 ± 28.0	145.6 ± 34.1	0.446
	DBP (mmHg)	77.8 ± 14.9	78.6 ± 18.5	0.204
	$\overset{.}{\mathrm{V}}{\mathrm{O}}_2$ (ml/min)	877.5 ± 426.2	902.4 ± 360.7	0.553
	$\overset{.}{\mathrm{V}}{\mathrm{O}}_2$ (ml/min/kg)	12.3 ± 5.5	13.9 ± 5.5	0.866
	${\overset{.}{\mathrm{V}}}_{\mathrm{E}}$ (ml/min)	32845.5 ± 15628.5	33580.0 ± 13222.2	0.398
	_Work load (watt)_	39.6 ± 27.9	59.6 **±** 32.1	0.312
Myokines	myostatin (ng/ml)	6.1 ± 2.0	9.0 ± 1.5	0.018
	IGF-1 (ng/ml)	219.7 ± 51.7	204.4 ± 59.6	0.176
	follistatin (ng/ml)	1042.7 ± 351.1	909.8 ± 409.0	0.398

Abbreviations: *SCI* spinal cord injury, *BMI* body mass index, *HR* heart rate, *SBP* systolic blood pressure, *DBP* diastolic blood pressure, *VO*_*2*_ oxygen uptake, *V*_*E*_, minute ventilation; *IGF-1* insulin-like growth factor-1

^a^by comparison between pre- and post-training groups using the Wilcoxon matched pairs test

## Discussion

The present study demonstrates that aerobic exercise training by arm ergometry results in increased serum myostatin level in patients with chronic SCI, even though the aerobic capacity and hemodynamic parameters do not change significantly. Acute exercise to exhaustion in the CPET leads to an immediate increase in serum IGF-1 in the reference group.

Myostatin has long been considered a cytokine that negatively regulates muscle growth. Recent evidence also point to its role in regulating the energy system of muscles. In the proposed “accelerator-brake” model, myostatin and IGF-1 act as counter-regulatory molecules for muscle hypertrophy [11–14, 20]. Thus, myostatin expression theoretically increases to limit the growth signals of muscle tissue such as exercise or growth factors.

On the other hand, animal studies suggest that myostatin is also related to aerobic capacity. *In vitro* study have shown that myostatin deficiency result in muscle fiber type conversion towards anaerobic metabolism [15, 16]. *In vivo*, there is also decreased exercise tolerance and oxygen consumption in myostatin-deficient mice [26]. Literature regarding responses of serum myostatin after exercise training in humans are limited and mainly focused on resistance training [27, 28]. Relevant data is limited for patients with SCI.

To date, this is the first study to show that aerobic exercise training by arm ergometry in patients with chronic SCI leads to elevated serum myostatin concentration. In the hypothesis of the “accelerator-brake” model [11–14, 20], IGF-1 increases first after exercise as muscle growth stimulus, followed by myostatin, which increases correspondingly to provide negative feedback signal. Our study failed to show increased IGF-1 after exercise training which may be due to inadequate exercise intensity, although further evidence is required to test the hypothesis. On the other hand, the increased myostatin may indicate an initial change of aerobic metabolism of muscle after aerobic training, although there are no detectable peak $\overset{.}{\mathrm{V}}{\mathrm{O}}_2$ changes in CPET.

The present study shows an immediate increase in serum IGF-1 level after acute exercise to exhaustion in CPET, but only in healthy participants and not in patients with SCI. IGF-1 is an age-related serum protein with growth controlling ability [17]. It affects the development of multiple systems, with the musculo-skeletal as one of the most important [18, 19]. Previous trials report an immediate increase in serum IGF-1 after bouts of exercise in healthy adults [29–31]. It was hypothesized that IGF-1 is released from skeletal muscle independent of growth hormone stimulation. The mean percentage of increase ranges from 13 % to 33 % in different studies [29–31], while the results here show an average of 18 % increase. Of note, the increase in IGF-1 is also influenced by exercise intensity [31]. All of the trials used an intensity of exercise training at or above AT $\overset{.}{\mathrm{V}}{\mathrm{O}}_2$. Part of the reason for the unresponsiveness of serum IGF-1 level after exercise in patients with SCI in the present study may be inadequate exercise intensity during CPET due to early fatigue.

There are few related studies available and a recent trial indicates no change in myostatin and IGF-I, as well as in body weight, among patients with SCI after chronic exercise training [21]. In contrast to the aerobic training by arm ergometry in this study, the cited trial used activity-based training that mainly consisted of resistance and locomotor training focused on the lower extremities.

The study had several limitations. Since this study did not measure the change in skeletal muscle mass or strength after exercise training, whether increased myostatin reflects a signal of muscle growth or a change in aerobic metabolism remains unanswered. Some of the patients with SCI in this study did not attain AT $\overset{.}{\mathrm{V}}{\mathrm{O}}_2$ during CPET due to early limb fatigue, and the true aerobic capacity may be underestimated. Similarly, the intensity of training (targeting an intensity of $\overset{.}{\mathrm{V}}{\mathrm{O}}_2$ at AT) may also be inadequate for most of the patients because of early limb fatigue. Furthermore, the reference group did not receive exercise training and as such, there is no comparison of training effect for patients with SCI. The limited case number due to difficulty in enrollment further lessens the discriminative power for the effect of exercise training. Moreover, there is a substantial heterogeneity in the SCI group, with varying severities and levels of injury, and large inter-individual differences in serum myokines. Further subgroup analysis is hampered by the small case number. Nevertheless, the results still show a marked increase in serum myostatin level after aerobic exercise training in chronic patients with SCI, although further studies are warranted to clarify the interaction between serum myostatin and changes in aerobic metabolism or muscle growth after training.

## Conclusions

This study evaluates the effects of aerobic exercise training by arm ergometry on serum concentrations of myokines in patients with chronic SCI. Acute exercise to exhaustion in CPET results in an immediate increase in serum IGF-1 in healthy individuals while aerobic exercise training results in increased serum myostatin in patients with SCI. The interaction between serum myostatin and changes in aerobic metabolism or muscle growth after training requires elucidation by further large-scale prospective studies.

## Abbreviations

SCI, spinal cord injury; CPET, Cardio-pulmonary exercise testing; $\overset{.}{\mathrm{V}}{\mathrm{O}}_2$, oxygen uptake; AT, anaerobic threshold; $\overset{.}{\mathrm{V}}{\mathrm{CO}}_2$, carbon dioxide production; IGF-1, insulin-like growth factor-1; ${\overset{.}{\mathrm{V}}}_{\mathrm{E}}$, minute ventilation; HR, heart rate; BP, blood pressure; ASIA, American Spinal Injury Association
